# Supplementary material for: CRISPR-Cas-based identification of a sialylated human milk oligosaccharides utilization cluster in the infant gut commensal Bacteroides dorei
Source: Nat Commun. 2024 Jan 2;15:105. doi: 10.1038/s41467-023-44437-y (PMC10761964; doi:10.1038/s41467-023-44437-y)
Supplement: Supplementary file 1 — Supplementary Information [file 41467_2023_44437_MOESM1_ESM.pdf]

Supplementary Figure 1

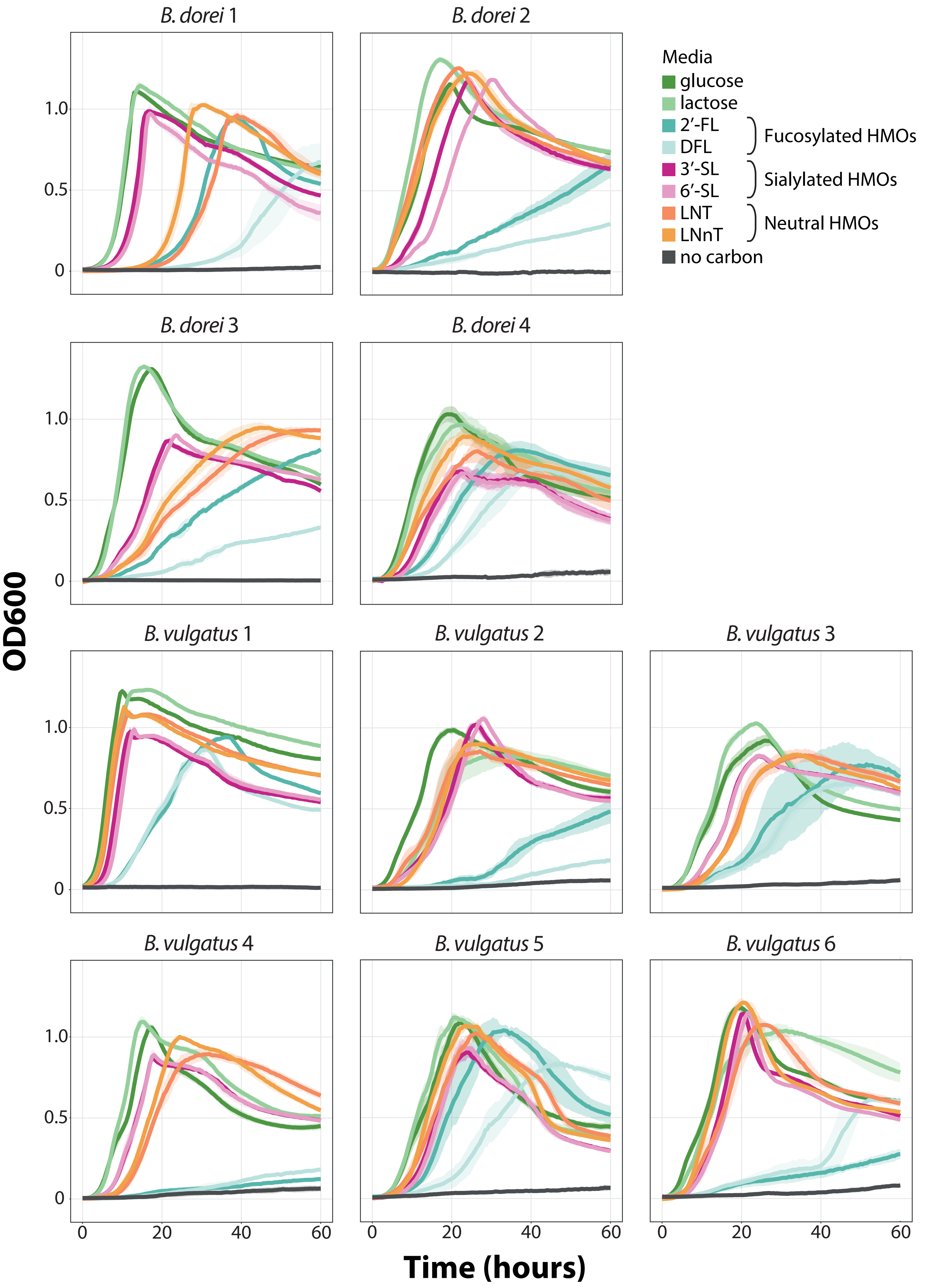

Supplementary figure 1. *B. dorei* and *B. vulgatus* grow on HMOs with varying efficiency. Growth curve plots of four *B. dorei* and six *B. vulgatus* strains on selected carbon sources (0.5% weight/volume): glucose, lactose, 2-Fucosyllactose (2'-FL), Difucosyllactose (DFL), 3-Sialyllactose (3'-SL), 6-Sialyllactose (6'-SL), Lacto-N-Tetraose (LNT) and Lacto-N-neotetraose (LNnT), no carbon media (negative control). Green shades represent positive control carbon sources, blues represent fucosylated HMOs, pinks represent sialylated HMOs and oranges represent neutral HMOs. Growth was averaged across two biological replicates, each consisting of three technical replicates (n = 6) and the lighter shade behind the growth curve represents the standard deviation. Specifically for *B. vulgatus* 3, the growth variability on 2'-FL was largely due to differences in the lag phase length and entry to the logarithmic phase between the biological replicates. Source data are provided as a Source Data file.

Supplementary Figure 2

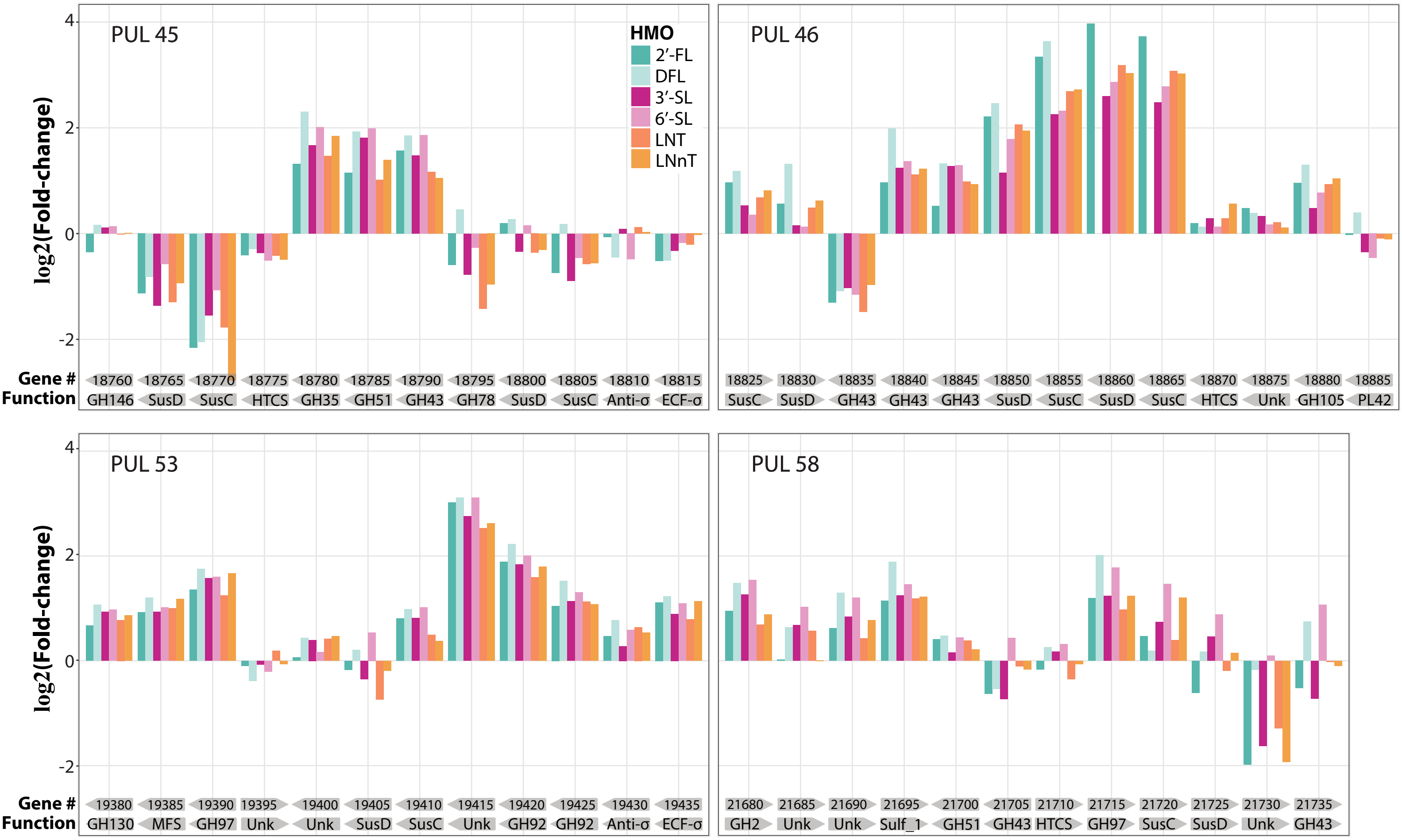

Supplementary figure 2. Differential expression of four additional PULs that do not change when growing on sialylated HMOs. Log2 fold-change of genes encoded in PUL 45, 46, 52 and 58 in *B. dorei*. Gene expression data in minimal media supplemented with HMOs (2′-FL, DFL, 3′-SL, 6′-SL, LNT, LNnT) was compared to gene expression on glucose, as in **Figure 1A**. The PULs presented here were arbitrarily selected. Source data are provided as a Source Data file.

Supplementary Figure 3

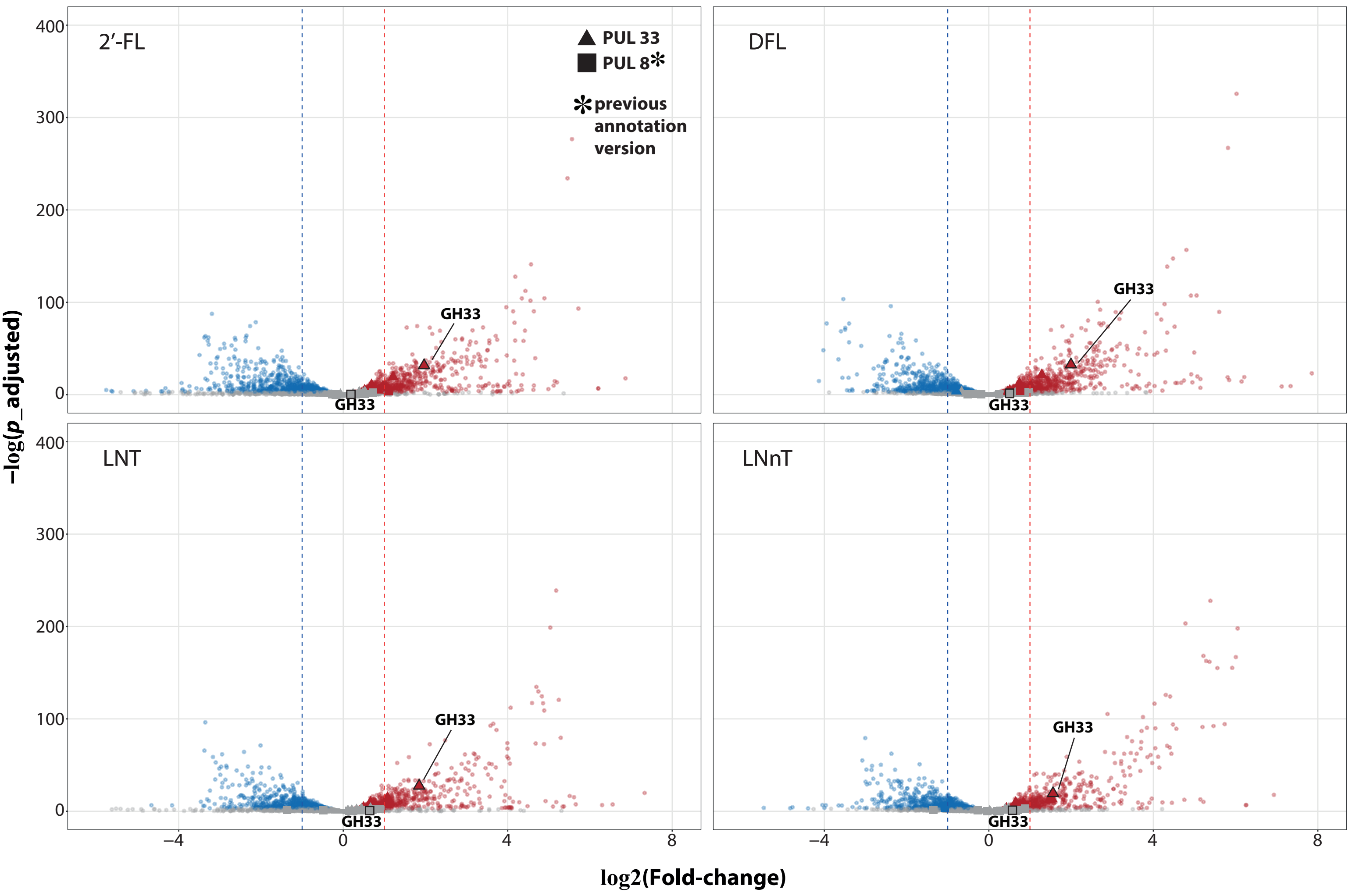

Supplementary figure 3. *B. dorei* does not upregulate PUL33 in response to fucosylated and neutral HMOs. Volcano plot, as in **Figure 1B**, of *B. dorei'*s gene expression in minimal media supplemented with fucosylated (2′-FL, DFL) and neutral (LNT, LNnT) HMOs compared to gene expression on glucose. Fold-change (log2, x-axis) of genes is plotted vs. the differential significance (-log2(p_adjusted), y-axis), with highlighted genes from PUL33 and the previous PULDB annotation of PUL8. GH: Glycoside hydrolase. Source data are provided as a Source Data file.

Supplementary Figure 4

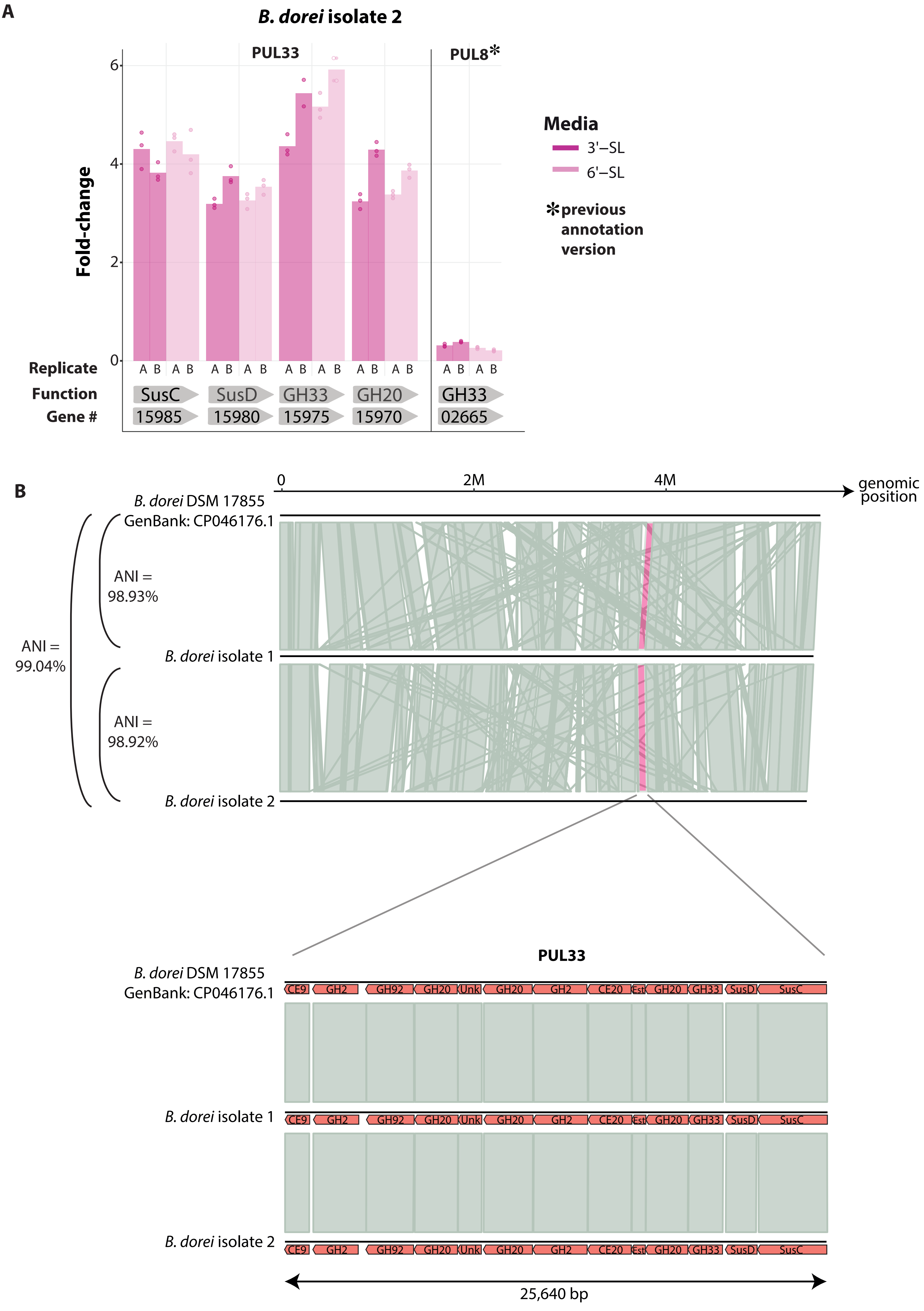

Supplementary figure 4. Comparison of two *B. dorei* isolates. (A) Reverse transcription quantitative PCR (RT-qPCR) data, depicting fold-change of gene expression comparing minimal media supplemented with sialylated HMOs (3'-SL, 6'-SL; dark and light pink, respectively) to glucose, for the isolate *B. dorei* 2. Fold-change was calculated using the efficiency-corrected ΔCq method[102]. The first four genes encoded in PUL33 are upregulated, whereas the GH33 gene from the previous PULDB annotation of PUL8 is not upregulated on sialylated HMOs, as was shown for *B. dorei* isolate 1 in **Figure 1A**. The RT-qPCR experiment was performed in two biological replicates (shown separately as A and B), each consisting of technical triplicates (n = 6). Individual data points are plotted and the bar represents their average. **(B)** Map of synteny regions across two *B. dorei* isolates and the type strain *B. dorei* DSM 17855 (top). The average nucleotide identity (ANI) are indicated. Zooming in to PUL33 shows the high conservation across strains of this region (bottom). SusC: SusC-like TonB-dependent transporter; SusD: SusD-like cell-surface glycan-binding protein; GH: Glycoside hydrolase; CE: Carbohydrate esterase; Est: Sialate O-acetylesterase; Unk: Unknown function. Source data are provided as a Source Data file.

Supplementary Figure 5

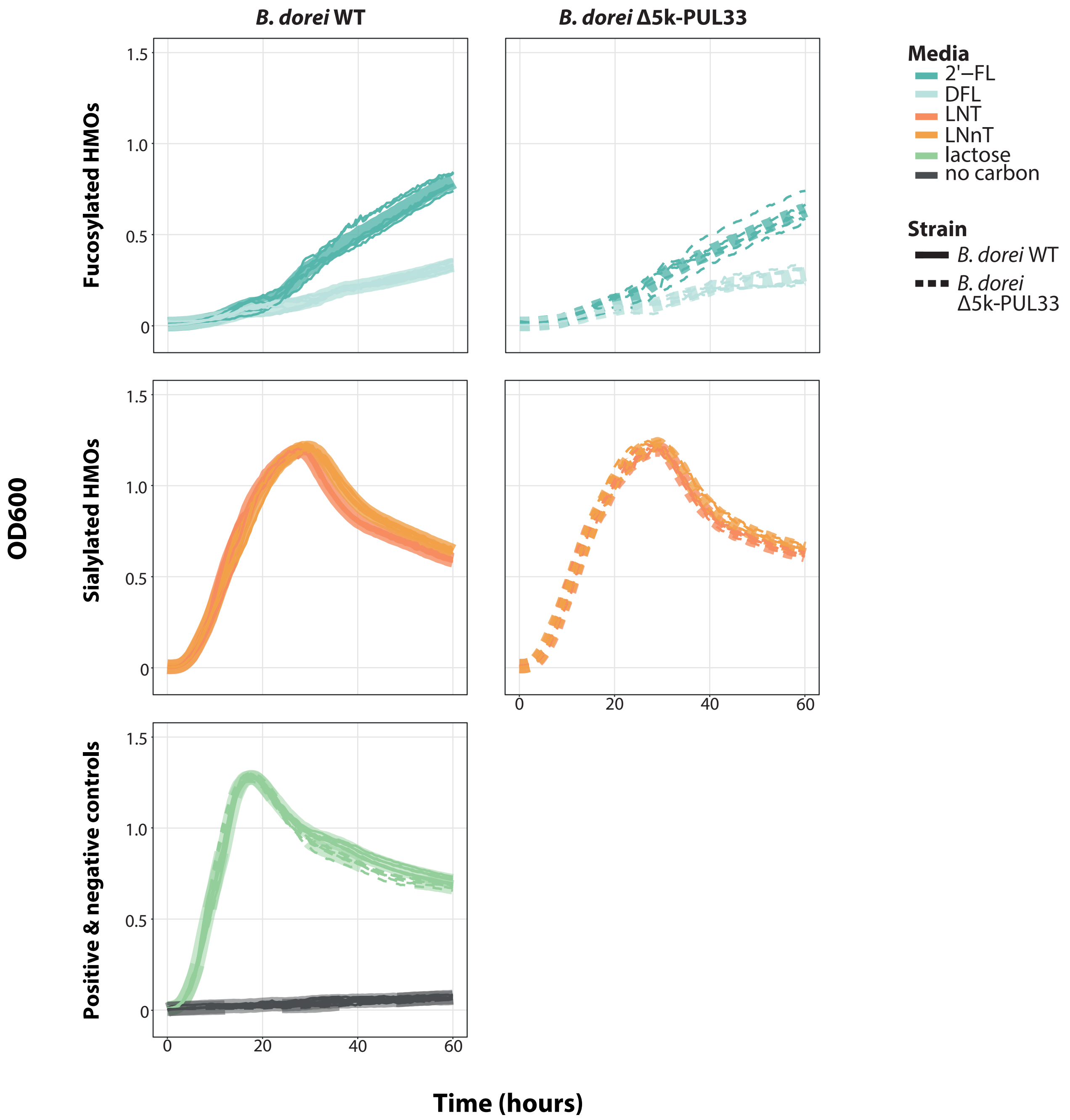

Supplementary figure 5. PUL33 is unnecessary for sialylated HMO utilization in *B. dorei*. Growth curve plots of *B. dorei* (solid line) and *B. dorei* Δ5k-PUL33 (dashed line) in minimal media supplemented with fucosylated HMOs (2'-FL, DFL; dark and light blue, respectively), neutral HMOs (LNT, LNnT; dark and light orange, respectively) HMOs, lactose (positive control; green) or no carbon media (negative control; gray), 0.5% weight/volume. Thick lines represent the average of two biological replicates, each consisting of three technical replicates, which are represented by thin lines (n = 6). Source data are provided as a Source Data file.

Supplementary Figure 6

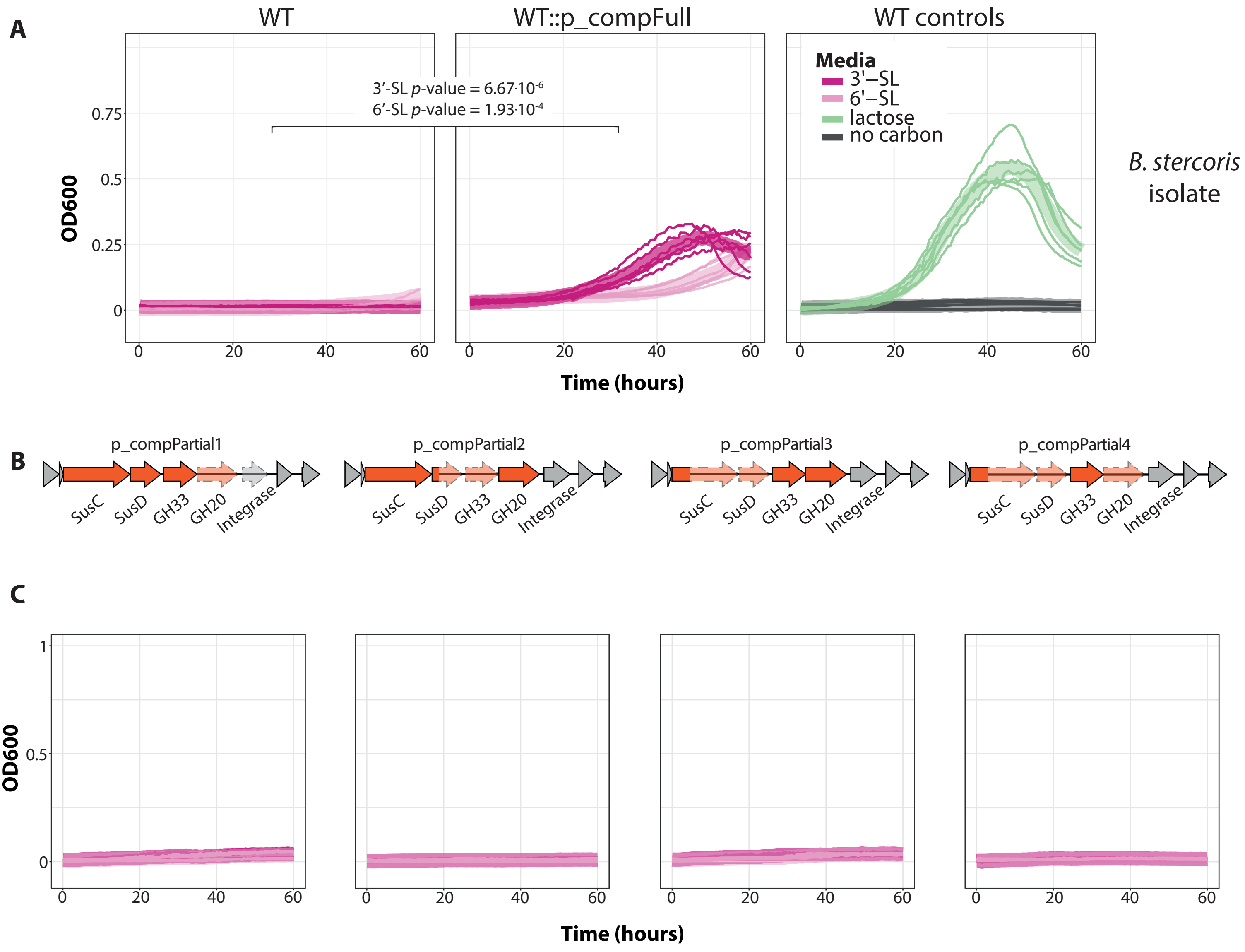

Supplementary figure 6. Complementation of genes from PUL33 in *B. stercoris*. (A) Growth curve plots of a *B. stercoris* isolate in minimal media supplemented with sialylated HMOs (3′-SL, 6′-SL; dark and light pink, respectively), glucose (green) or water (no carbon; gray), 0.5% weight/volume. Thick lines represent the average of two biological replicates, each consisting of three technical replicates, which are represented by thin lines (n = 6). *p*-values were calculated using a paired two-sided t-test at 50 h. This strain gained the ability to grow on sialylated HMOs once conjugated with p_compFull. **(B)** Structure overview of the plasmids p_compPartial1/2/3/4, as in **Figure 3B**. **(C)** Growth curve plots of a *B. stercoris* isolate complemented with p_compPartial1/2/3/4, in minimal media supplemented with sialylated HMOs (3′-SL, 6′-SL), 0.5% weight/volume. The colors and replicates are the same as in **(A)**. This strain was not able to utilize sialylated HMOs following conjugation with all partial plasmids tested. Source data are provided as a Source Data file.

Supplementary Figure 7

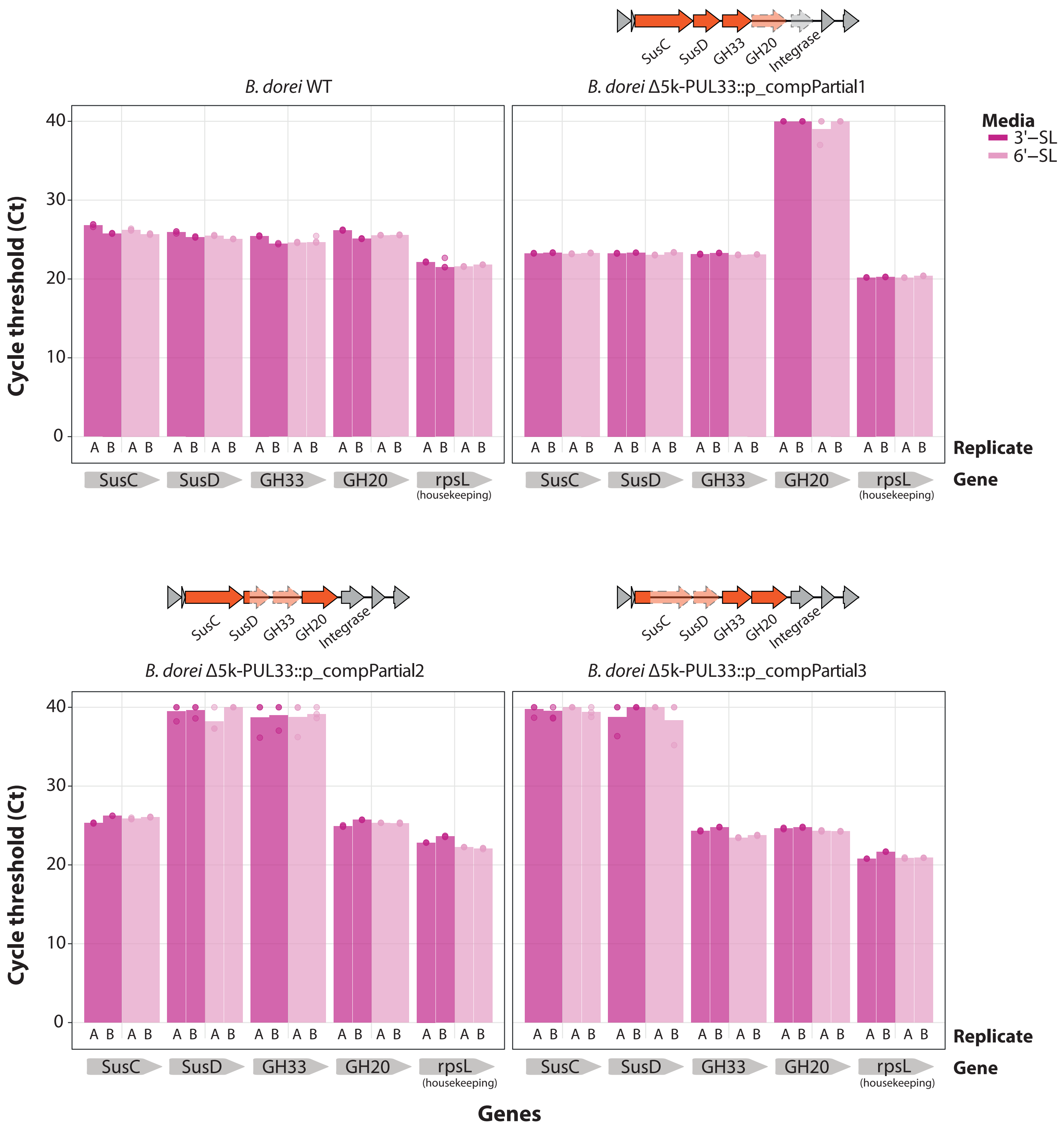

Supplementary figure 7. Complemented genes are expressed in transconjugated mutants. Reverse transcription quantitative PCR (RT-qPCR) results for the first four genes encoded in PUL33 (SusC, SusD, GH33, GH20) and the housekeeping gene rpsL in *B. dorei* wild-type (WT) and *B. dorei* Δ5k-PUL33 complemented with the plasmids p_compPartial1/2/3. Gene expression was measured when growing on sialylated HMOs (3′-SL and 6′-SL, dark and light pink, respectively) compared to glucose. A high (>35) cycle threshold (Ct) value represents a gene that is not expressed, whereas lower Ct values indicate the gene is expressed under the conditions tested. The RT-qPCR experiment was performed in two biological replicates (shown separately as A and B), each consisting of technical triplicates (n = 6). Individual data points are plotted and the bar represents their average. Source data are provided as a Source Data file.

Supplementary Figure 8

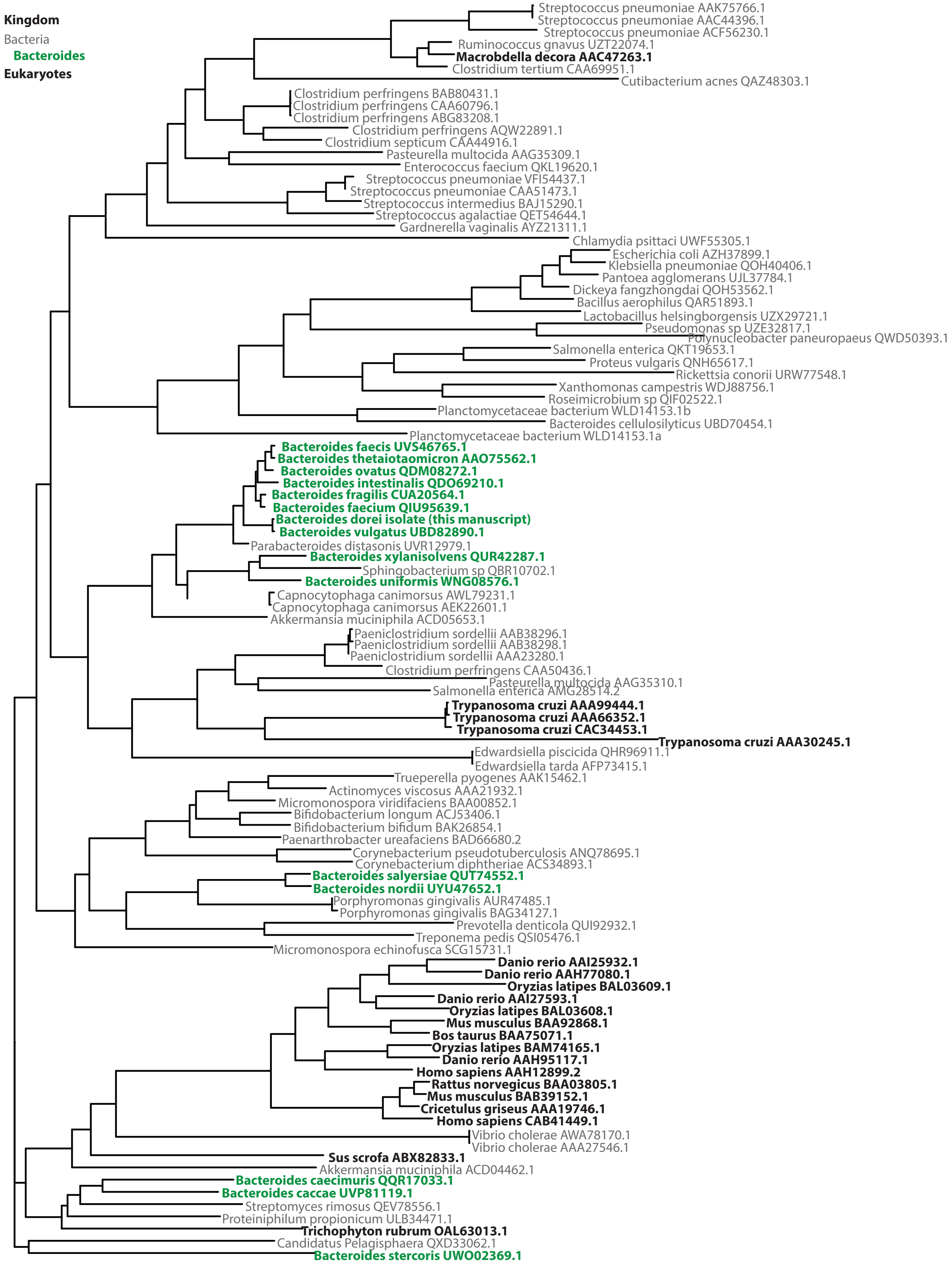

Supplementary figure 8. Phylogenetic tree of GH33 protein sequences. Organism names are indicated to the species level, next to the GenBank accession number of the GH33 protein analyzed. The tree was generated using the previously published SACCHARIS pipeline.

Supplementary Figure 9

A

GH33

	B. dorei isolate	*B. vulgatus* ATCC 8482 (UBD82890.1)	*B. thetaiotaomicron* VPI 5482 (AAO75562.1)
B. dorei isolate	100 %		
B. vulgatus ATCC 8482 (UBD82890.1)	97.44 % (0.064 Å)	100 %	
B. thetaiotaomicron VPI 5482 (AAO75562.1)	67.65 % (0.444 Å)	67.46 % (0.458 Å)	100 %

B

GH33

```
  B. dorei   MRNPSLLLLIVVFILAPFKLSAAADTVAVRETRIPVLIERQDNELFHLRIEAAQSQMLNEVKLDFGKDVNLNEIESVKLYYGGTESVERRGKTYFAPVDYISNNTPGKTLAANTSYSVLK  120
B. vulgatus  MRNPSLLLLIVVFILAPFKLSAAADTVVVRETRIPVLIERQDNELFHLRIEATQSQMLNEVKLDFGKDVNLNEIESVKLYYGGTESVERRGKTYFAPVDYISNNTPGKTLAANTSYSVLK  120
  B. theta   MKRNHYL-FTLILLLGCSIFVKASDTVFVHQTQIPILIERQDNVLFYFRLDAKESRMMDEIVLDFGKSVNLSDVQAVKLYYGGTEALQDKGKKRFAPVDYISSHRPGNTLAAIPSYSIKC  119
             *:.   * : ::::*.   :  *:*** *::*:**:******* **::*::* :*:*::*: *****.***.::::*********::: :**. ********.: **:****  ***:

  B. dorei   SEVKAPKREVILKADQKLFPGVNYFWISLQMKPIASILSKVSAKVVEAKIDGQIAPLKI-VRKADTHYMGVGVRHAGDDGAAAYRIPGLVTSNKGTLLGVYDVRYNNSADLQEYVEIGLS  239
B. vulgatus  SEVKAPKREVVLKADQKLFPGVNYFWISLQMKPVASILSKVSAEVVEAKIDGQVAPLKI-ARKADTHYMGIGVRHAGDDGAAAYRIPGLATSNKGTLLGVYDVRYNNSADLQEYVEIGLS  239
  B. theta   AEALQPSAKVVLKSHYKLFPGINFFWISLQMKPETSLFTKISSELQSVKIDGKEAICEERSPKDIIHRMAVGVRHAGDDGSASFRIPGLVTSNKGTLLGVYDVRYNNSVDLQEYVDVGLS  239
             :*.  *. :*:**:. *****:*:********* :*::*:*:: ..****: *  :    *   * *.:*********:*::*****.****************.*.******::***
                                                                                                         RIP motif

  B. dorei   RSTDGGQTWEKMRIPMAFGEYDGLPKAQNGVGDPAILVDKKTGTIWIVAAWTHGMGNGRAWWNSQTGMDRNHTAQLMMVKSDDDGKTWSEPMNITEQVKDPSWYFLLQGPGRGISMEDGT  359
B. vulgatus  RSTDGGQTWEKMRIPMAFGEYDGLPKAQNGVGDPAILVDKKTGTIWIVAAWTHGMGNGRAWWNSQTGMDRNHTAQLMMVKSDDDGKTWSEPMNITEQVKDPSWYFLLQGPGRGISMEDGT  359
  B. theta   RSTDGGKTWEKMRLPLSFGEYDGLPAAQNGVGDPSILVDTQTNTIWVVAAWTHGMGNQRAWWSSHPGMDLYQTAQLVMAKSTDDGKTWSKPINITEQVKDPSWYFLLQGPGRGITMSDGT  359
             ******:******:*::******** ********:****.:*.***:********** ****.*: ***  :****:*.** *******:*:**********************:*.***
             ASP box #1                                                                                    ASP box #2

  B. dorei   LVFASQYIGSDRIPNAGVIYSKDHGKTWNISTLARTNTTESQVVEVEPGVLMLNMRDNRGGSRAVSTTTDLGKTWKEHESSRTALQEPVCMASLISVKAKENVLGKDILLFSNPNDTKNR  479
B. vulgatus  LVFASQYIGNDRIPNAGIIYSKDHGKTWNISTLARTNTTESQVAEVEPGVLMLNMRDNRGGSRAVSTTTDLGKTWKEHESSRTALQEPVCMASLISVKAKENVLGKDILLFSNPNDAKNR  479
  B. theta   LVFPTQFIDSTRVPNAGIMYSKDRGKTWKMHNMARTNTTEAQVVETEPGVLMLNMRDNRGGSRAVAITKDLGKTWTEHPSSRKALQEPVCMASLIHVEAEDNVLDKDILLFSNPNTTRGR  479
             *** :*:*.. *:****::****:****::  .:*******:**.*.*******************: *.******.** ***.************ *:*::***.********** ::.*
                                     ASP box #3

  B. dorei   HSITIKASLDGGVTWLPENQLLLDAGWGWGYSCLTMIDKETVGILYESSVAHMTFQAIKLKDIVKTK  546
B. vulgatus  HSITIKASLDGGVTWLPENQLLLDAGWGWGYSCLTMIDKETVGILYESSVAHMTFQAIKLKDIIKTK  546
  B. theta   NHITIKASLDDGLTWLPEHQLMLDEGEGWGYSCLTMIDRETIGILYESSAAHMTFQAVKLKDLIR--  544
             : ********.*:*****:**:** * ***********:**:*******.*******:****:::
                  ASP box #4                  catalytic residues
```

C

SusC

	B. dorei isolate	*B. vulgatus* ATCC 8482 (ABR41747.1)	*B. thetaiotaomicron* VPI 5482 (AAO75559.1)
B. dorei isolate	100 %		
B. vulgatus ATCC 8482 (ABR41747.1)	98.26 %	100 %	
B. thetaiotaomicron VPI 5482 (AAO75559.1)	20.67 %	20.58 %	100 %

Supplementary figure 9. Comparison of GH33 and SusC protein sequences in *B. dorei*, *B. vulgatus*, and *B. thetaiotaomicron*. **(A)** Distance matrix, with distances calculated using the HHalign algorithm, comparing the protein sequence of the GH33 sialidase gene in our *B. dorei* isolate, *B. vulgatus* ATCC 8482 and *B. thetaiotaomicron* VPI 5482. GenBank accessions of the protein sequences are indicated under the strain name in brackets. The root mean square deviation (RMSD) value is presented within the matrix in brackets, for an alignment of 521 out of 546 residues for all three proteins (see **Methods**). **(B)** Multiple sequence alignment of GH33, with structural elements of sialidases (RIP motif, ASP boxes) shaded in gray, catalytic and other residues essential for activity shaded in red and residues participating in the formation of the wide-open groove around the active site shaded in pink. **(C)** Distance matrix as in **(A)**, of the SusC transporter protein sequences of our *B. dorei* isolate, *B. vulgatus* ATCC 8482 and *B. thetaiotaomicron* VPI 5482.

Table S1. Growth curve statistical data. Values in bold are also reported in the main figures.

B. dorei

			Paired t-test		
Comparison	Time	Media	mean difference	p-value	
WT vs Δ5k-PUL32	1h	3'-SL	-0.007	0.004636	
		6'-SL	0.003	0.2809	
	24h	3'-SL	1.012533	**5.07E-08**	Fig. 2E
		6'-SL	0.8990667	**1.30E-07**	Fig. 2E
Δ5k-PUL32 vs Δ5k-PUL32::p_compFull	1h	3'-SL	5.00E-04	0.8022	
		6'-SL	1.83E-03	0.3759	
	24h	3'-SL	0.7543333	**9.55E-07**	Fig. 2E
		6'-SL	0.4895	**1.64E-11**	Fig. 2E

B. uniformis

			Paired t-test		
Comparison	Time	Media	mean difference	p-value	
WT vs WT:: p_compFull	1h	3'-SL	-0.001666667	0.5483	
		6'-SL	-0.002666667	0.02929	
	50h	3'-SL	0.3986667	**4.98E-10**	Fig. 3A
		6'-SL	0.4351667	**3.39E-07**	Fig. 3A

B. stercoris

			Paired t-test		
Comparison	Time	Media	mean difference	p-value	
WT vs WT:: p_compFull	1h	3'-SL	0.0255	0.0009527	
		6'-SL	-0.001666667	0.6431	
	50h	3'-SL	0.2646667	**6.67E-06**	Fig. 3A
		6'-SL	0.1636667	**1.93E-04**	Fig. 3A

Table S2. Bacterial strains and plasmids used in this study.

Strain or Plasmid name	Sequence description	Source	Notes
**Parental strains**			
E. coli S17-1 λ pir	thi pro hdsR hdsM+ recA, chromosomal insertion of RP4-2(Tc::Mu Km::Tn7) , AmpS	Ilan Rosenshine's lab	
B. uniformis CL03T12C37		Naama Geva Zatorsky's lab	
B. dorei 1		This study	Isolated from a breastfeeding infant stool sample
B. dorei 2		This study	
B. stercoris		This study	
**Deletion strains**			
B. dorei Δ5k-PUL32		This study	
**Complemented strains**			
B. dorei Δ5k-PUL32::p_compFull		This study	
B. dorei Δ5k-PUL32::p_compPartial1		This study	
B. dorei Δ5k-PUL32::p_compPartial2		This study	
B. dorei Δ5k-PUL32::p_compPartial3		This study	
**Deletion plasmids**			
pB006	AmpR-ermG-p134-TetR-R6k-oriT	Zheng et al. 2022	Replicon plasmid in B. thetaiotamicron
pB036	p134-P1TDPGH023-FnCas12a-P1-gRNA (gRNA-AGCTTGTGTGGAATATACTCCGTCA)	Zheng et al. 2022	aTc-inducible FnCas12a expression, gRNA expressed from pB006 backbone. Was used for a 5k bp deletion of the RGI-PUL in B. thetaiotamicron
p_KO	p134-P1TDPGH023-FnCas12a-P1-gRNA (gRNA-CGCCATGATATGCAAAGTCCGGAAG)	This study	aTc-inducible FnCas12a expression, gRNA expressed from pB006 backbone.
**Complementation plasmids**			
pNBU2_erm	pNBU2-ErmG-AmpR	Koropatkin et al. 2008	Integrates cloned fragments to NBU2 att1 and/or att2 sites
pNBU2_erm_P5E1	pNBU2-ErmG-AmpR-P5E1	Bencivenga-Barry et al. 2020	pNBU2_erm backbone, expression of genes under *B. fragilis*' P5E1 promoter

p_compFull	pNBU2_erm_P5E1-SusC-SusD-GH33-GH20	This study	
p_compPartial1	pNBU2_erm_P5E1-SusC-SusD-GH33	This study	
p_compPartial2	pNBU2_erm_P5E1-SusC-GH20	This study	
p_compPartial3	pNBU2_erm_P5E1-GH33-GH20	This study	

Table S3. Primers and genetic tools used in this study.

Primer Number	Primer Name	Sequence 5'-3'	Comments
**Primers for KO validation**			
P1		CAAAGACAGTGGTAAACATCC	
P2		GAAAGATAAGCTGACTCGTTC	
P3		CGGATTGGTAAACTCCACCT	
P4		ATAATGAAACGCTGGTTTTC	
**Primers for p_KO construction**			
P5	AmplifyBackbone_F	GATCTTCCGGGGGCTTTCTCATGCGTTGGATCC**CTTCCGGACTTTGCATATCATGGCG**ATCTACAAGAGTAGAAATTaac	pB036 from Zheng et al. 2022 was used as a template. gRNA in bold
P6	AmplifyBackbone_R	aattccgaacaactggag	
P7	AmplifyLF_F	CATGAGAAAGCCCCCGGAAGATCACCTTCCGGGGGCTTTTTTATTGCGCCGCGTCTCAGCCATCTTAAACTTACCG	
P8	AmplifyLF_R	CAGATGAGGTGGAGTTTACCAATCCGC	
P9	AmplifyRF_F	GATTGGTAAACTCCACCTCATCTGTGTTCACATTCCCATTAAAAGGC	
P10	AmplifyRF_R	gcctccagttgttcggaattCGACGAACCGTAAGACTTCG	
**Primers for p_compFull construction**			
P11	Amplify4Genes_F	AACATTTAAAAAATAACATtccATGAAGAAAGCTCTCTTTTTTATCGTATGCCTC	Gibson assembled with the SalI & ncoI digested pNBU2_erm_P5E1 from Bencivenga-Barry et al. 2020
P12	Amplify4Genes_R	ACTGGAAGATAGGCAATTAGTTATTTTTGTTTATCTGGTGAATAAATCAATCCTTCTACCGCC	
**Primers for p_compPartial1 construction**			
P13		CTAATTGCCTATCTTCCAGT	p_compFull was used as the template
P14		TATTTATTTGGTCTTTACAATATC	
**Primers for p_compPartial2 construction**			
P15		ATAATGAAACGCTGGTTTTC	p_compFull was used as the template
P16		AATATTTATAATTTTACGGGTGT	
**Primers for p_compPartial2 construction**			
P17		ATTTTTGTCTGATAAGTATCC	p_compFull was used as the template
P18		ACTTTCTCTTTTTTACATATTAAAATGAC	
**Primers for RT-qPCR**			

P19	Dorei_rpsL_F	CGTGTGCGTCTGACTAACTC	Based on Ben-Assa et al. 2020
P20	Dorei_rpsL_R	GTGATAACGTACACCCGGAA	
P21	SusC_F	GGCACGGGCACCATAAATAG	
P22	SusC_R	CAAGAATCTGGAAGGTCCGT	
P23	SusD_F	AGTCCAGATTGCTACGACTG	
P24	SusD_R	CAAAGGCTGCCACATTGATG	
P25	GH33_F	GGTTCCACTTCCACTACCTG	
P26	GH33_R	TCGTATTCCTAATGCCGGTG	
P27	GH20_F	TCCAATTGCCGAAGTGTCTG	
P28	GH20_R	GAAGCTAGGTTAAACCGGGA	

**Genetic tool**	**Description**	Sequence 5'-3'	Comments
guide RNA	Used for a 5kb knock-out in *B. dorei*, with the p_KO plasmid	TTCTCTAATCCGAATGATACCAAGA	
		ATGAGCATCTATCAAGAATTTGTGAATAAATATAGTCTTAGTAAAACATTGAGATTCGAACTGATCCCGCAAGGAAAAACTCTGGAAAATATTAAAGCACGTGGATTGATTTTGGATGATGAAAAACGTGCTAAAGATTATAAAAAAGCCAAACAGATAATTGATAAATATCATCAATTCTTCATTGAAGAAATCCTTAGTAGTGTATGCATTAGCGAAGATCTGCTTCAGAATTATAGTGATGTATATTTTAAACTGAAAAAATCTGATGATGATAATTTACAGAAAGACTTTAAATCAGCTAAAGATACAATAAAGAAACAAATTAGTGAATATATTAAAGATAGTGAAAAGTTTAAAAATTTGTTTAACCAGAATCTGATCGATGCTAAAAAAGGACAGGAATCCGATCTGATTCTGTGGTTGAAACAGAGTAAAGATAATGGTATTGAATTGTTTAAAGCCAATTCTGATATCACTGACATTGATGAAGCACT	
FnCas12a		GGAAATTATCAAATCCTTTAAAGGTTGGACTACCTATTTTAAAGGTTTTCACGAAAATCGTAAAAATGTGTATTCTAGTAATGATATACCGACGTCGATTATCTATCGTATCGTAGATGATAATCTGCCGAAATTTCTGGAAAATAAAGCTAAATACGAATCTCTGAAAGATAAAGCACCCGAAGCCATCAATTATGAACAGATTAAGAAGGATCTGGCTGAAGAACTGACATTTGACATAGATTATAAAACGTCTGAAGTAAATCAGCGCGTATTTAGTCTGGATGAAGTGTTCGAAATCGCAAATTTTAACAATTACTTAAATCAGTCGGGTATAACCAAGTTTAATACCATAATCGGAGGAAAATTTGTAAATGGAGAAAATACGAAACGCAAAGGGATCAATGAATATATCAATTTGTATTCTCAGCAGATTAACGATAAAACATTGAAAAAATATAAAATGAGTGTACTGTTCAAACAAATCCTGTCTGATACCGAATCTAAAAGTTTCGTAATCGATAAATTAGAAGATGATTCTGATGTGGTGACTACAATGCAGAGCTTCTATGAACAGATCGCTGCATTCAAAACTGTAGAAG	
		AAAAGAGTATTAAGGAAACACTGTCTTTGCTGTTTGATGATCTGAAAGCTCAGAAACTTGACTTAAGCAAAATCTACTTCAAAAACGATAAAAGTCTGACTGATTTAAGCCAGCAAGTTTTCGACGACTATAGTGTAATTGGCACCGCCGTATTGGAATATATCACTCAGCAAATTGCCCCTAAAAATCTGGATAACCCGTCGAAGAAAGAACAGGAACTGATTGCCAAGAAAACCGAAAAGGCTAAATATCTGAGCTTGGAAACAATCAAACTCGCACTGGAAGAATTCAATAAGCATCGGGATATTGATAAACAATGTCGTTTTGAAGAAATTCTGGCCAATTTTGCTGCAATTCCTATGATAT	

		TCGATGAAATTGCACAAAATAAAGATAACTTAGCCCAGATTTCTATTAAATATCAGAACCAGGGTAAAAAAGATCTTTTGCAGGCGAGTGCCGAAGATGATGTAAAGGCTATTAAAGATCTGCTGGATCAAACTAATAATTTGTTACATAAACTGAAGATTTTCCATATCTCGCAGTCTGAAGATAAAGCTAATATTTTGGATAAAGACGAACATTTCTATCTGGTATTTGAAGAATGTTATTTCGAATTAGCAAACATTGTTCCGCTGTATAACAAAATTCGTAATTATATTACTCAAAAACCGTATTCAGATGAAAAATTTAAACTGAATTTTGAAAATAGTACATTAGCTAACGGATGGGATAAGAATAAAGAACCTGATAATACAGCGATTTTATTCATCAAAGACGATAAATACTATTTGGGAGTTATGAATAAGAAAAATAATAAAATCTTTGATGATAAAGCTATTAAAGAAAATAAAGGAGAAGGTTATAA	
		GAAAATAGTTTATAAATTGCTGCCAGGAGCTAATAAGATGCTGCCTAAAGTATTTTTCAGTGCTAAATCTATTAAATTCTATAACCCGTCAGAGGACATCTTAAGAATTCGGAATCATAGCACCCATACAAAGAATGGATCTCCCCAAAAAGGATATGAAAAGTTCGAATTTAATATTGAAGATTGCAGAAAATTTATTGATTTCTATAAACAATCTATTAGTAAACATCCCGAATGGAAAGATTTCGGCTTTCGTTTCTCTGATACACAAAGATATAATAGTATTGATGAATTTTATAGAGAAGTAGAAAATCAGGGCTATAAGCTGACGTTCGAAAATATTTCAGAATCCTATATAGATAGTGTAGTTAATCAGGGTAAATTGTATTTATTTCAGATTTATAATAAAGACTTTTCTGCCTATTCTAAAGGACGTCCTAATCTGCATACACTCTATTGGAAAGCTTTATTCGATGAACGTAACCTCCAAGATGTAGTAT	
		ATAAGCTCAATGGAGAAGCAGAATTGTTCTATCGCAAGCAAAGTATTCCTAAAAAGATCACTCATCCTGCTAAAGAAGCCATTGCTAATAAGAATAAAGATAACCCTAAGAAAGAATCAGTATTTGAATATGATTTGATAAAAGATAAACGTTTCACTGAAGATAAGTTTTTCTTCCATTGTCCGATAACAATTAACTTTAAGTCCAGTGGAGCAAATAAATTCAATGATGAAATCAATCTGCTTCTTAAAGAAAAAGCAAATGATGTACATATTTTGTCTATTGATAGAGGTGAACGTCACCTTGCATATTATACTTTAGTGGATGGAAAGGGAAATATCATCAAGCAGGATACCTTTAATATTATCGGAA	
		ATGACCGTATGAAAACGAACTATCATGATAAATTGGCTGCTATTGAAAAGGATCGTGATAGCGCTCGTAAAGACTGGAAGAAAATTAATAATATCAAAGAAATGAAAGAGGGATATCTGTCGCAGGTAGTACACGAAATCGCTAAACTGGTAATTGAATATAATGCCATCGTAGTATTTGAGGATCTGAATTTCGGTTTCAAGCGTGGACGTTTTAAAGTAGAAAAGCAAGTATATCAGAAATTAGAAAAGATGTTAATTGAAAAATTAAATTATCTGGTTTTCAAAGATAATGAATTCGATAAAACCGGAGGCGTCTTGAGAGCCTATCAGTTAACTGCCCCTTTTGAAACTTTTAAAAAGATGGGTAAACAGACAGGAATTATTTATTATGTGCCGGCCGGATTTACATCTAAAATCTGCCCGGTGACTGGTTTCGTGAACCAACTGTATCCGAAATATGAAAGTGTTAGCAAATCCCAGGAATTCTT	
		TAGCAAATTTGATAAAATATGTTATAATTTGGATAAAGGATATTTTGAATTCAGTTTTGACTATAAAAATTTTGGAGATAAAGCCGCAAAGGGTAAGTGGACTATTGCTAGTTTCGGATCGCGCTTGATTAATTTTCGTAATTCAGATAAAAACCATAATTGGGATACTCGTGAAGTGTATCCTACCAAAGAACTGGAAAAATTGTTAAAGGACTATAGTATTGAATATGGACATGGTGAATGTATAAA	

		AGCAGCAATATGCGGAGAAAGTGATAAGAAATTCTTTGCAAAATTGACTAGCGTACTGAATACTATATTGCAAATGCGTAATAGTAAAACAGGAACAGAACTGGATTATCTGATCAGCCCTGTAGCAGACGTAAACGGCAATTTCTTTGATAGTCGCCAAGCTCCTAAAAATATGCCGCAAGACGCTGATGCAAATGGAGCATATCACATTGGTCTTAAGGGACTGATGCTGCTGGGTCGCATTAAAAATAATCAGGAAGGTAAAAAACTGAACTTGGTAATCAAAAATGAAGAATATTTTGAATTCGTACAGAATCGTAATAATTAA	
Bf_P5E1 constitutive promoter + ribosome binsing site (RBS)	Used for gene complementation assays	CTCTAGATAAAACGAAAGGCTCAGTCGAAAGACTGGGCCTTTCGTTTTACAATTGGGCTACCTTTTTTTTGTTTTGTTTGCAATGGTTAATCTATTGTTAAAATTTAAAGTTTCACTTGAACTTTCAAATAATGTTCTTCTATTTGCAGTGTCGAAAGAAACAAAGTAGGACTGATCACACAACATTTAAAAAATAACAT	
