# Supplementary material for: CRISPR-Cas-based identification of a sialylated human milk oligosaccharides utilization cluster in the infant gut commensal Bacteroides dorei
Source: Nat Commun. 2024 Jan 2;15:105. doi: 10.1038/s41467-023-44437-y (PMC10761964; doi:10.1038/s41467-023-44437-y)
Supplement: Supplementary file 3 — Reporting Summary [file 41467_2023_44437_MOESM3_ESM.pdf]

nature portfolio

Corresponding author(s): *Double-anonymous peer review submissions: write DAPR and your manuscript number here instead of author names.*

Last updated by author(s): *YYYY-MM-DD*

Reporting Summary

Nature Portfolio wishes to improve the reproducibility of the work that we publish. This form provides structure for consistency and transparency in reporting. For further information on Nature Portfolio policies, see our Editorial Policies and the Editorial Policy Checklist.

Statistics

For all statistical analyses, confirm that the following items are present in the figure legend, table legend, main text, or Methods section.

n/a	Confirmed	
☐	☒	The exact sample size (*n*) for each experimental group/condition, given as a discrete number and unit of measurement
☐	☒	A statement on whether measurements were taken from distinct samples or whether the same sample was measured repeatedly
☐	☒	The statistical test(s) used AND whether they are one- or two-sided<br>*Only common tests should be described solely by name; describe more complex techniques in the Methods section.*
☒	☐	A description of all covariates tested
☐	☒	A description of any assumptions or corrections, such as tests of normality and adjustment for multiple comparisons
☐	☒	A full description of the statistical parameters including central tendency (e.g. means) or other basic estimates (e.g. regression coefficient) AND variation (e.g. standard deviation) or associated estimates of uncertainty (e.g. confidence intervals)
☐	☒	For null hypothesis testing, the test statistic (e.g. *F*, *t*, *r*) with confidence intervals, effect sizes, degrees of freedom and *P* value noted<br>*Give P values as exact values whenever suitable.*
☒	☐	For Bayesian analysis, information on the choice of priors and Markov chain Monte Carlo settings
☒	☐	For hierarchical and complex designs, identification of the appropriate level for tests and full reporting of outcomes
☒	☐	Estimates of effect sizes (e.g. Cohen's *d*, Pearson's *r*), indicating how they were calculated

Our web collection on statistics for biologists contains articles on many of the points above.

Software and code

Policy information about availability of computer code

Data collection	No software was used to collect data. Optical density data were collected using Biotek plate reader. All data used in this study was generated by the Yassour lab, and sequencing was performed in the Hebrew University of Jerusalem.
Data analysis	Optical density data, outputted by the Epoch2 microplate spectrometer, was parsed and analyzed using R v4.2.3. RNA-seq data was analyzed using Trim Galore v0.6.6 for read filtering, Bowtie2 v2.4.5 for mapping, featureCounts v2.0.3 and DESeq v1.41.0 for differential expression analysis. Nanopore reads of whole genome sequencing data were analyzed with Guppy v6.1.2 (ONT) for basecalling, Nanoplot v1.33.1 for quality assessment, Porechop v0.2.4 for read filtering, Flye v2.9 for assembly. Synteny maps of isolates were produced using the gggenomes R package v0.9.5.9000. Plots were generated using the ggplot2 R package v3.4.2. The phylogenetic tree of GH33 sequences was generated using the previously published SACCHARIS pipline version 1.0. Protein visualization and alignment was performed using PyMOL version 2.5.4.

For manuscripts utilizing custom algorithms or software that are central to the research but not yet described in published literature, software must be made available to editors and reviewers. We strongly encourage code deposition in a community repository (e.g. GitHub). See the Nature Portfolio guidelines for submitting code & software for further information.

Data

Policy information about availability of data

All manuscripts must include a data availability statement. This statement should provide the following information, where applicable:

- Accession codes, unique identifiers, or web links for publicly available datasets
- A description of any restrictions on data availability
- For clinical datasets or third party data, please ensure that the statement adheres to our policy

The RNA-seq data used in this study is available in the NCBI database under SRA accession codes SRS11934615 (HMOs) and SRS11934616 (glucose). The Nanopore sequencing whole genome assembly data for B. dorei isolates generated in this study have been deposited in the NCBI database under GenBank assembly accessions GCA_030122685.1 (B. dorei 1) and GCA_030122665.1 (B. dorei 2).

Research involving human participants, their data, or biological material

Policy information about studies with human participants or human data. See also policy information about sex, gender (identity/presentation), and sexual orientation and race, ethnicity and racism.

Reporting on sex and gender	We collected samples from a total of 10 infants.. No sex and gender reporting or analysis was performed in this study. We isolated strains from their stool samples, and did not use any corrections for sex or gender.
Reporting on race, ethnicity, or other socially relevant groupings	No ethnicity information was collected from the participants.
Population characteristics	All infants were less than six months old, were breastfed and did have never used antibiotics. The breastfeeding infant stool samples were used for strain isolation only and no statistical inferences (where covariants would be relevant) were made based on them.
Recruitment	Mothers saw our ad on social media, and then were approached with details about the study and provided a single stool sample of their child. Our method of participant selection might introduce a self-selection bias, however no comparison was performed between the infants participating in the study, or between the infants participating in this study to other cohorts. The stool samples were merely used for strain isolation of Bacteroides species for exploration of their HMO utilization strategies, hence breastfeeding was the most important constraint when recruiting infants.
Ethics oversight	The Hebrew University, Institutional Review Board approval number 20042021.

Note that full information on the approval of the study protocol must also be provided in the manuscript.

Field-specific reporting

Please select the one below that is the best fit for your research. If you are not sure, read the appropriate sections before making your selection.

- [x] Life sciences
- [ ] Behavioural & social sciences
- [ ] Ecological, evolutionary & environmental sciences

For a reference copy of the document with all sections, see nature.com/documents/nr-reporting-summary-flat.pdf

Life sciences study design

All studies must disclose on these points even when the disclosure is negative.

Sample size	10 stool sample. No sample size calculation was performed as no statistical analysis was performed based on these stool samples, and they were used for strain isolation. The manuscript essentially focuses on two strains of B. dorei that were isolated from the stool samples and describes a biological pathway that was discovered using basic science microbiology methods of genetic manipulation. Hence, sample size and statistical force considerations are less relevant in this context.
Data exclusions	All data were used.
Replication	For all growth curve experiments, experiments were performed using two biological replicates, in triplicates (n = 6), and were repeated twice. Replication attempts were successful.
Randomization	None. Randomization and covariant control is irrelevant in the contex of the work performed in this manuscript, as described above. No comparison between samples was made. The manuscript essentially focuses on two strains of B. dorei that were isolated from the stool samples and describes a biological pathway that was discovered using basic science microbiology methods of genetic manipulation.
Blinding	We performed all data generation without known which sample is which.

Reporting for specific materials, systems and methods

We require information from authors about some types of materials, experimental systems and methods used in many studies. Here, indicate whether each material, system or method listed is relevant to your study. If you are not sure if a list item applies to your research, read the appropriate section before selecting a response.

Materials & experimental systems

n/a	Involved in the study
☒	☐ Antibodies
☒	☐ Eukaryotic cell lines
☒	☐ Palaeontology and archaeology
☒	☐ Animals and other organisms
☒	☐ Clinical data
☒	☐ Dual use research of concern
☒	☐ Plants

Methods

n/a	Involved in the study
☒	☐ ChIP-seq
☒	☐ Flow cytometry
☒	☐ MRI-based neuroimaging

Plants

Seed stocks	*Report on the source of all seed stocks or other plant material used. If applicable, state the seed stock centre and catalogue number. If plant specimens were collected from the field, describe the collection location, date and sampling procedures.*
Novel plant genotypes	*Describe the methods by which all novel plant genotypes were produced. This includes those generated by transgenic approaches, gene editing, chemical/radiation-based mutagenesis and hybridization. For transgenic lines, describe the transformation method, the number of independent lines analyzed and the generation upon which experiments were performed. For gene-edited lines, describe the editor used, the endogenous sequence targeted for editing, the targeting guide RNA sequence (if applicable) and how the editor was applied.*
Authentication	*Describe any authentication procedures for each seed stock used or novel genotype generated. Describe any experiments used to assess the effect of a mutation and, where applicable, how potential secondary effects (e.g. second site T-DNA insertions, mosiacism, off-target gene editing) were examined.*
